# Derivation and Characterization of Novel Cytocompatible Decellularized Tissue Scaffold for Myoblast Growth and Differentiation

**DOI:** 10.3390/cells13010041

**Published:** 2023-12-24

**Authors:** Anshuman Singh, Suraj Kumar Singh, Vinod Kumar, Jalaj Gupta, Manoj Kumar, Devojit Kumar Sarma, Samradhi Singh, Manoj Kumawat, Vinod Verma

**Affiliations:** 1 Stem Cell Research Centre, Department of Hematology, Sanjay Gandhi Post Graduate Institute of Medical Sciences, Lucknow 226014, India; 2 National Institute of Animal Biotechnology (NIAB), Hyderabad 500032, India; 3 ICMR—National Institute for Research in Environmental Health, Bhopal 462030, Indiadevojit.sarma@icmr.gov.in (D.K.S.); samradhi.singh@icmr.gov.in (S.S.);

**Keywords:** cellular agriculture, decellularization, mushroom scaffold, myoblast, cultured meat

## Abstract

The selection of an appropriate scaffold is imperative for the successful development of alternative animal protein in the form of cultured meat or lab-grown meat. Decellularized tissues have been suggested as a potential scaffold for cultured meat production owing to their capacity to support an optimal environment and niche conducive to cell proliferation and growth. This approach facilitates the systematic development of 3D tissues in the laboratory. Decellularized scaffold biomaterials have characteristics of high biocompatibility, biodegradation, and various bioactivities, which could potentially address the limitations associated with synthetic bio-scaffold materials. The present study involved the derivation and characterization of a decellularized scaffold from mushroom tissue following subsequent assessment of the scaffold’s capacity to support myogenic differentiation. Mushroom sections were soaked in nuclease and detergent solution for 4 days. Furthermore, decellularization was confirmed by histology and DAPI staining, which showed the removal of cellular components and nuclei. Myoblast cells were seeded onto decellularized tissue, which exhibited excellent cytocompatibility and promoted myogenic growth and differentiation. The study’s findings can serve as a foreground for the generation of an edible and natural scaffold for producing a safe and disease-free source of alternative animal protein, potentially reducing the burden on the health sector caused by conventional animal protein production and consumption.

## 1. Introduction

The production and consumption of conventional animal protein sources such as animal meat have emerged as a potent risk factor for various zoonotic and chronic diseases, including COVID-19, cardiovascular disease, diabetes, cancer, stroke, etc. [1,2,3,4], thus raising concern for human health. Over-use of antibiotics in livestock feeding and healthcare is also one of the prominent health risk factors. Moreover, inappropriate use of antibiotics in animal farming influences people’s health via the food chain [5]. Moreover, conventional meat production demands a significant amount of agricultural land, energy, and clean water for raising and maintaining livestock for meat production purposes; in addition, the enormous release of greenhouse gases into the environment from unutilized digestive nutrients constitutes a significant challenge to the world [6]. Cellular agriculture, categorized into two major subfields, i.e., cultured meat and fermentation products, has been suggested as a potential alternative to conventional animal protein sources [7]. Cultured meat has the potential to be a disease-free and healthier alternative animal protein source [8]. Furthermore, cultured meat production could address the environmental health issues associated with conventional meat production, such as the annual emission of a large amount of greenhouse gases (GHGs), the consumption of approximately 8% of global freshwater, and the use of 30% of land for livestock farming [9,10]. Also, livestock farming raises ethical concerns about animal welfare; thus, cultured meat may become competitive in terms of production costs and animal ethics when compared to conventional meat [11].

Cultured meat or in vitro meat production involves the cultivation and growth of myotubes onto a bioengineered scaffold [6]. Notably, scaffolds determine the structure and texture of cultured meat, which is vital for the shelf-life and nutritional content of cultured meat [12,13]. Scaffolds can be molded into desirable shapes that provide physical support and an appropriate niche-resembling environment for growth for muscle cell anchorage [14,15]. Decellularized tissue scaffold possesses immense potential for the development of alternative animal proteins. Decellularization involves the removal of cellular and nuclear components, but the 3D structure and extracellular matrix part of the tissue are preserved [16,17,18]. In previous studies, various decellularized plant tissue (including spinach leaves, endosperm, apple hypanthium, broccoli florets, etc.)-based scaffolds have been reported for cultured meat production [19]. Plant tissues are abundant, easy to obtain, and economically cheap, and decellularized plant tissue scaffolds stimulate growth, proliferation, and differentiation [14,20]. Therefore, decellularized plant-based scaffolds have been proposed as natural and edible cell carriers for affordable scaled cultured meat production systems, which contribute to the nutritional properties of cultured meat [21]. However, the presence of anti-nutrients in plant tissue, such as protease inhibitors, amylase inhibitors, lectins (phytohemagglutinin), polyphenols (particularly tannins), and phytic acid poses a serious health risk and adverse physiological effects such as altered gut function and endocrine disruption, among others [22,23].

Considering the high nutritional and nutraceutical properties and their peculiar structural compositions, mushrooms could be a promising scaffold for growing disease-free alternative animal protein in the laboratory. Mushrooms are a significant source of essential nutrients, including carbohydrates, dietary fibers, vitamins (B1, B2, B12, C, D, and E), minerals, and proteins [24]. Also, mushrooms exhibit antibacterial, immune system enhancer, and cholesterol-lowering properties and are thus useful for promoting human health [25]. Mushroom scaffolds provide different topography, porosity, and pore size for culturing mammalian cells for tissue-engineering applications [26]. Previously, the potential of decellularized mushroom tissue scaffold was investigated for stem cell-based bone tissue regeneration [27]. Thus, the present study highlights the potential of decellularized mushrooms as an edible scaffold for myoblast growth and differentiation. The developed decellularized mushroom scaffold (DMS) exhibited excellent compatibility with myoblast cells without any detrimental impacts on cells. The outcome of the study offers a novel approach to developing a natural, sustainable, and cytocompatible scaffold to produce a cost-effective and disease-free alternative source of animal protein in the form of cultured meat.

## 2. Materials and Methods

### 2.1. Materials

For C2C12 mouse myoblast cell culture, DMEM/F12 medium (Gibco; cat no. 11330-032), fetal bovine serum (FBS; Gibco, Waltham, MA, USA; cat no. 10270-106), non-essential amino acid (Gibco; cat no. 11140050), penicillin-streptomycin (Gibco; cat no. 15140122) were used. Button mushroom (*Agaricus bisporus*) was procured from the local supermarket. Furthermore, the mushroom was decellularized by sodium deoxycholate (Sigma, St. Louis, MO, USA; cat no. 30970), DNase (Thermo Scientific, Waltham, MA, USA; cat no. EN0525), and RNase (Thermo Scientific; cat no. R1253). Hematoxylin (SRL, Mumbai, India; cat no. 40362) and eosin Y (SRL; cat no. 29391) were used for the characterization of decellularization.

### 2.2. Cell Culture

C2C12 mouse myoblast cells were cultured in DMEM/F12 medium supplemented with 15% FBS, 1× non-essential amino acid, penicillin-streptomycin at 37 °C, and 5% CO_2_ incubator. C2C12 myoblast cells were allowed to grow at 100% confluency for spontaneous differentiation into myotubes [28], which were characterized by assessing the expression of the myosin heavy chain (*MHC*) gene.

### 2.3. Decellularization of Mushroom Tissue

Thin circular (10 mm) sections of mushroom cap were formed using the serological blade and biopsy punch. Sections of mushroom were collected into 1× PBS supplemented with 1× penicillin-streptomycin, followed by rinsing with 1× PBS twice. Furthermore, sections of the mushroom were incubated in a decellularization solution (2% sodium deoxycholate, 140 U/mL DNase, and 100 U/mL RNase) for 4 days at room temperature. After 4 days, tissue sections were removed from the decellularization solution, washed thoroughly with 1× PBS thrice, and stored at 4 °C till further use.

### 2.4. Characterization of Decellularization in Mushroom Tissue

Decellularized mushroom tissues were initially characterized by hematoxylin and Eosin (H&E) staining; native mushroom tissue sections were used as control. Tissue sections were fixed in 10% neutral-buffered formalin for 24 h, followed by dehydration in 70% ethanol. Tissue sections were embedded in paraffin and sectioned at 2.5 µm thickness with a microtome. Paraffin from the tissue samples was removed in three changes of xylene (2 min per change). Tissue samples were hydrated by transferring the slides through three changes of 100%, 95%, and 70% ethanol for 2 min per change. Slides were rinsed in running tap water at room temperature for 2 min. Tissue sections were stained with a hematoxylin solution for 3 min followed by thorough rinsing with distilled water for 5 min. Furthermore, the sections were stained with eosin Y for 2 min, dehydrated in 95% ethanol for 20 min, and then transferred in 95% and 100% ethanol for 2 min each, followed by three changes of xylene each for 2 min. Sections were mounted using glycerol, and images were captured. Furthermore, the characterization of the removal of DNA from decellularized tissues was performed by DAPI staining. Mushroom tissue sections (decellularized and native both) were rinsed with 1× PBS twice and fixed in 4% paraformaldehyde (Invitrogen, Waltham, MA, USA; cat no. FB0002) for 30 min, followed by washing with 1× PBS once. Tissue sections were incubated in 1 µg/mL DAPI solution for 10 min. in the dark at room temperature. After 10 min incubation, tissue sections were rinsed thoroughly with 1× PBS thrice and mounted in prolonged gold antifade reagent (Invitrogen; cat no. P10144). Images were captured under a Zeiss confocal microscope.

### 2.5. DNA Isolation and Quantification

Native and decellularized mushroom tissues were blot-dried, and dry weight was recorded. Tissues were properly homogenized in 100 µL TE buffer (1 M Tris HCl, 0.5 M EDTA) followed by 600 µL lysis buffer (100 mM Tris HCl, 10 mM EDTA, 2% SDS), mixed by inversion, and incubated for 30 min at 65 °C. Lysis buffer was centrifuged at 13,000 rpm at 4 °C for 15 min; in the supernatant, add 400 µL chloroform: isoamyl alcohol (24:1) and centrifuged at 13,000 rpm at 4 °C for 15 min. The aqueous phase was pipetted out in fresh tubes, and DNA was precipitated by adding the 100% ethanol and centrifuged at 16,000 rpm at 4 °C for 15 min. Ethanol was dried, and DNA was resuspended in 20 µL nuclease-free water. The quality (A260/280) and quantity of DNA were recorded by spectrophotometer.

### 2.6. Biodegradation Analysis of DMS

Decellularized and native mushroom scaffolds were washed with 1xPBS and incubated in lysozyme solution (100 µg/mL) at 37 °C. The initial dry weight of the scaffold was noted as W_I_. The scaffolds incubated with lysozyme at 37 °C were sampled after 1, 3, and 7 days. The dry weight of the degraded sample was noted as W_F_, and the percentage of degradation was calculated as:

% biodegradation = 100 × (W_I_ − W_F_)/W_I_

### 2.7. Cytocompatibility of DMS

The cytocompatibility of DMS was assessed by performing the DAPI staining of seeded cells, and the viability of the seeded myoblast cells on the DMS was assessed by performing the live-dead staining using Calcein AM and propidium iodide (PI). Before cell seeding, mushroom scaffolds were sterilized using 70% ethanol for 30 min, followed by thorough washing with 1xPBS thrice. Monolayer myoblast cells were used as control. Furthermore, 5 × 10^5^ myoblast cells were seeded onto DMS. After 72 h, cells were washed with 1× DPBS and stained with 1 µg/mL DAPI for 10 min. Cells were rinsed with 1× DPBS thrice, mounted with prolonged gold antifade reagent (Invitrogen; cat no. P10144), and captured under a Zeiss confocal microscope.

For viability (live/dead staining), 5 × 10^5^ myoblast cells were seeded onto DMS, and after 72 h, monolayer myoblast and cell-seeded DMS were rinsed with 1× PBS followed by incubation with a staining solution containing 2 µM Calcein AM and 1 µM PI for 30 min. in the dark at room temperature; after 20 min incubation, 1 µg/mL DAPI was added to the staining solution. After incubation, the staining solution was removed, and monolayer myoblast cells and scaffolds were rinsed with 1× PBS and mounted with a prolonged gold antifade reagent (Invitrogen; cat no. P10144). Images were captured under the Zeiss confocal microscope (Jena, Germany).

### 2.8. In Vitro Myogenesis on DMS

Myogenesis of myoblast cells seeded onto DMS was investigated by the trichrome staining following the protocol of Valderrama and colleagues [29] with slight modification. Scaffold sections were sterilized in 70% ethanol for 30 min, followed by thorough washing with 1× PBS. A total of 5 × 10^5^ myoblast cells were seeded onto DMS for 72 h. Monolayer myoblast cells were used as control. Thereafter, monolayer myoblast and cell-seeded DMS were fixed in Bouin’s solution (2.1% picric acid, 40% formaldehyde, 5% glacial acetic acid) overnight. The next day, the fixative solution was removed, and scaffolds were washed with 1× PBS once and stained in hematoxylin solution for 2 min, followed by thorough rinsing with distilled water for 10 min. Furthermore, they were stained with a mixture of 0.5% erythrosine B and 0.5% orange G for 30 min at room temperature, followed by a quick rinse in distilled water. Later, they were immersed in 0.5% phosphotungstic acid for 10 min, rinsed with distilled water, and dehydrated in three consecutive baths of 95% and 100% ethanol. Finally, the cell-seeded DMS was cleaned with xylene and mounted using glycerol. Images were captured under an inverted phase contrast microscope.

### 2.9. Gene Expression Analysis of Differentiated Myoblast Clusters on DMS

Gene expression analysis of myoblast marker *MyoG* and differentiated myoblast marker *MHC* was performed by semi-quantitative PCR. RNA isolation of monolayer differentiated myoblast and cluster grown on the scaffold was performed by the Trizol method. Reverse transcription of RNA into cDNA was performed using the Superscript III First-Strand Synthesis Kit (Invitrogen; Cat no.-18080051). Primers used for PCR are listed in Table 1. PCR was performed using the HotStar Taq Plus Mater Mix kit (Qiagen, Venlo, The Netherlands; cat no. 203643). The PCR product was visualized at 1.2% agarose gel. Quantification of relative expression of the *MYH* gene was performed by Image J software (ImageJ 1.53t) by normalizing the band intensity value of the *GAPDH* with the *MYH* gene of each group.

### 2.10. Statistical Analysis

Data are presented as the mean with standard error bars representing the standard deviation. Data were analyzed by a two-sample Student *t*-test with equal variance for significance and considered significantly different if *p* < 0.05.

## 3. Results

**Figure 1:**
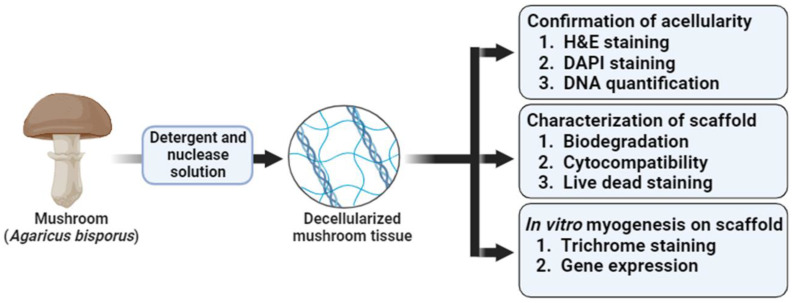
Schematic presentation of the study.

Decellularized tissues provide the ECM framework to the harvested cells, which, in turn, maintains the native environment of tissues, promotes cell attachment, growth, and proliferation, and provides a niche for cell proliferation and differentiation [30]. Decellularized tissues have been applied to bone, skin tissue engineering and grafting, blood arteries, heart valves, and kidney bladders, among other tissue-engineering applications [31]. The decellularized mushroom scaffold used in the present study has been shown to provide ECM-like support for the growth and differentiation of myoblast cells, which indicates its scaffolding potential for the development of an alternative disease-free animal protein source (Figure 1).

### 3.1. Generation and Characterization of Decellularized Mushroom Tissue

**Figure 2:**
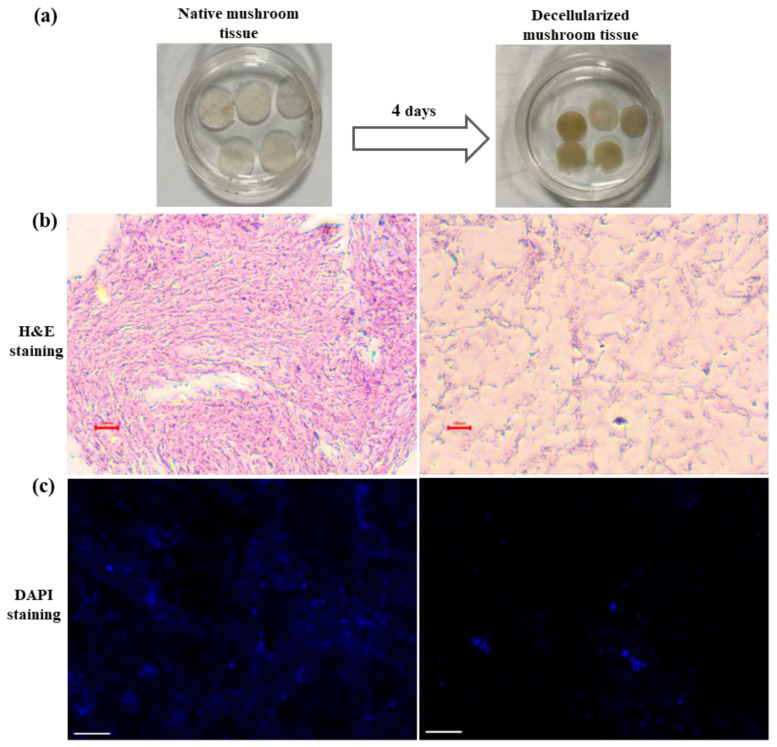
Analysis of decellularization via histological analysis and DNA quantification. (**a**) Decellularization of mushroom tissue. (**b**) Decellularization was analyzed by performing the H&E (scale 100 µm) and (**c**) DAPI staining showing the acellularity in decellularized tissue (scale 100 µm).

**Figure 3:**
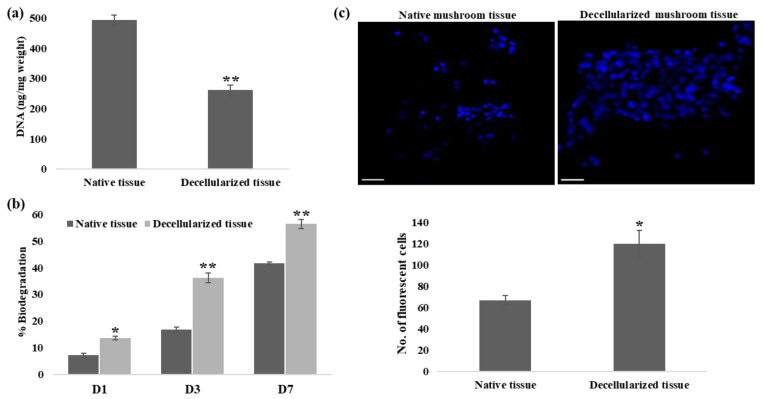
Characterization and cytocompatibility of decellularized mushroom tissue. (**a**) Quantification of DNA in native and decellularized tissue (*p*-value 0.005). (**b**) Biodegradation kinetics of native and decellularized mushroom tissue (*p*-value 0.01 on D1, 0.006 on D3, and 0.007 on D7). (**c**) Evaluation of cytocompatibility of native and decellularized mushroom tissue scaffold with myoblast cells using DAPI staining showing better cytocompatibility of the decellularized mushroom tissue (*p*-value 0.028) scale 100 µm. Data are reported as mean ± SD. * *p* < 0.05, ** *p* < 0.01.

Mushroom tissue sections were exposed to a decellularization solution for 4 days; decellularized mushroom sections shrunk, and their color changed from white to dark brown (Figure 2a). Furthermore, histological analysis by H&E staining was used for the characterization of acellularity in decellularized tissue. H&E-stained native mushroom tissue displayed intact tissue possessing blue and pink-stained nuclei and cellular material, respectively, while the absence of blue and pink color from decellularized tissue confirms the elimination of cellular components (Figure 2b). In addition, qualitative confirmation of decellularization was performed by assessing the presence of DNA by DAPI staining for nuclei [32]. DAPI staining illustrated the absence of nuclei and the removal of DNA following the decellularization of mushroom tissue (Figure 2c). In continuation, quantification of DNA showed that decellularized tissues possessed statistically lower DNA (~50% removal of DNA) as compared to native tissue (Figure 3a).

### 3.2. Evaluation of Scaffold Properties of Decellularized Mushroom Tissue: Biodegradation and Cytocompatibility

The degradation profile of the scaffold is one of the key attributes of scaffold used for alternative animal protein production. Degradation produced by lysozymes at 37 °C is a standard assay to compare scaffolds that will be used to culture cells [33]. Decellularized mushroom tissues showed significantly higher degradation than native tissue on days 1, 3, and 7 (Figure 3b). Decellularized tissue showed approximately 13%, 36%, and 57% degradation in comparison to approximately 7%, 17%, and 41% degradation of native tissue on days 1, 3, and 7, respectively, which clearly indicates the excellent biodegradation properties of decellularized mushroom tissues. Furthermore, cytocompatibility of the scaffold (adherence and growth of myoblast cells on decellularized mushroom tissue) was confirmed by performing the DAPI staining. Quantification of fluorescence signals illustrated the significantly higher cell attachment on decellularized tissue in comparison to native tissue. Results demonstrate that decellularized mushroom tissue possessed high cytocompatibility with myoblast cells (Supplementary Figure S1). Moreover, DMS showed excellent cytocompatibility with goat dermal fibroblast cells (Figure 3c and Supplementary Figure S2); the result indicates the potential of DMS to be utilized for alternative animal protein production by seeding the livestock cells.

**Figure 4:**
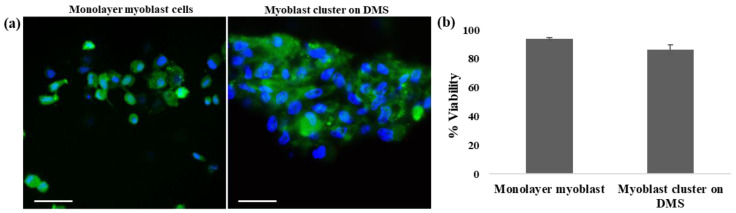
Viability assessment of the myoblast cells seeded on mushroom tissue scaffold. (**a**) Calcein AM/PI staining of myoblast cells in monolayer and clusters on DMS (scale 100 µm). (**b**) Quantification showed the non-significant difference in %viability of the myoblast cells in monolayer and cluster on DMS (*p*-value 0.06). Data are reported as mean ± SD.

In addition, the viability of the myoblast cells was assessed by performing the live-dead assay using Calcein AM/PI staining (Figure 4 and Supplementary Figure S3). Calcein AM passively crosses the cell membrane and, in the cytosol, is hydrolyzed by the enzyme esterase to a polar green-fluorescent product, and thus a green signal of Calcein AM represents the viable cells [34] while PI exerts red color to the dead cells. Myoblast clusters grown on decellularized tissue showed similar viability in comparison to monolayer myoblast cells (Figure 4a,b).

### 3.3. In Vitro Myogenesis on DMS

**Figure 5:**
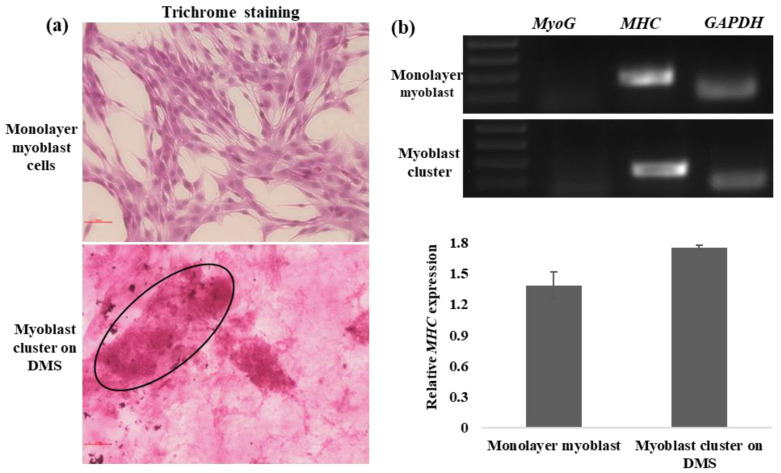
In vitro myogenesis on DMS. (**a**) Trichrome staining showed the red-stained monolayer myoblast and cluster on DMS (circle) (scale 100 µm). (**b**) Comparative expression of the myogenesis-specific genes (*MyoG* and *MHC*) and (**c**) Relative expression of the *MHC* gene in monolayer myoblast and myoblast cluster on DMS (*p*-value 0.054). Data are reported as mean ± SD.

The early stage of in vitro myogenesis of myoblast cells exhibits rounded morphology, which further establishes cell-cell connections in the form of cell clusters [33,35]. Likewise, we also observed that myoblast cells seeded onto DMS formed cell clusters (after 72 h of seeding), which indicates the efficacy of our scaffold towards myogenesis. Moreover, trichrome staining was performed to validate the early myogenic-promoting potential of DMS [33]. Trichrome staining specifically exerts a red color to the myogenic lineage; likewise, large and intense red-stained myoblast clusters were observed on DMS (Figure 5a), which highlights the potential of the developed scaffold in myogenesis. Furthermore, monolayer myoblast cells showed the expression of *MHC*, a marker of differentiated myoblast cells, and similar expression of the *MHC* gene was observed in myoblast clusters harvested from DMS confirms that the clusters represent the early myogenesis and differentiation into myotubes (Figure 5b,c).

## 4. Discussion

With the advent of cellular agriculture, it is possible to produce alternative animal protein through the cultivation of stem cells derived from myotubes onto a scaffold under controlled physiological conditions [36]. Cellular agriculture gives rise to a safe and disease-free animal protein source and, in this way, overcomes the blooming burden on the health sector caused by the production and consumption of conventional meat. Notably, the scaffold provides a 3D microenvironment to the growing cells, and for cultured meat application, the scaffold should be edible, sustainable, widely available, animal-free, non-toxic, and can provide nutritional value to the end product [37]. Decellularized tissue scaffolds are gaining attention for alternative animal protein production, and various studies have used decellularized plant tissue scaffolds for the cultivation and growth of different cell types. Decellularized apple hypanthium tissue-derived cellulose scaffold supports the adherence and proliferation of mouse myoblast cells [16]. Jones and colleagues have grown and assessed the viability, differentiation, and alignment of the seeded bovine satellite cells on a decellularized spinach scaffold for laboratory-grown meat application [38]. In addition, Thyden and colleagues reported that decellularized broccoli floret supported adherence and viability of bovine satellite cells in suspension, thus suggesting a novel, edible scaffold for lab-grown meat [21]. Moreover, decellularized spinach scaffolds have been shown to increase the osteogenic differentiation of bone marrow-derived human mesenchymal stem cells (BM-MSCs) [39]. However, it is suggested that characterization of decellularized tissue for complete cell removal is the key step for further application of decellularized tissue scaffold as residual cellular material diminishes the constructive tissue remodeling advantages of the decellularized biologic scaffold materials and thus affects the efficacy of the decellularized tissue scaffold [40,41]. In previous studies, the characterization of decellularized tissue for complete cell removal is performed by H&E and DAPI staining [42]. H&E staining of the native mushroom tissue shows blue and pink-stained nuclei and cellular components, respectively, while in decellularized mushroom tissues, no or minimal cellular component was visualized, thus indicating the acellularity of the mushroom tissue. In accordance with previous studies, very little blue fluorescence of DAPI staining and significantly lower DNA quantity (~50% removal of DNA) in decellularized mushroom tissue confirms the removal of DNA and acellularity in decellularized mushroom tissue [43]. Like previous studies, results indicate that detergent and nuclease-based approach decellularizes the mushroom tissue [27,44,45,46]; however, further refinement would lead to the development of more efficient decellularization protocol for mushroom tissue.

Furthermore, cytocompatibility assessment of the decellularized tissue scaffold is one of the important concerns of the scaffolds [47]. Previously, various biofunctionalized decellularized plant tissues (leaves, stem) from different plant species were tested for the expansion of human mesenchymal stem cells (MSCs), dermal fibroblast cells, umbilical vein endothelial cells [48,49]. For alternative animal protein production, the scaffold should be compatible with myotubes, adipocytes, etc., so that it can recapitulate the conventional meat. Decellularized mushroom tissue shows excellent cytocompatibility with different cell types, differentiated myoblast cells, and fibroblast cells in comparison to native mushroom tissue and, thus, could emerge as a potent scaffold material for alternative animal protein production. Furthermore, vi120hability assessment using Calcein AM/PI staining shows that the growing clusters of differentiated myoblast cells possess similar viability with monolayer myoblast cells; ≥93% viability in monolayer myoblast while ≥84% viability in myoblast clusters after 3 days of seeding onto DMS.

In addition, for alternative animal protein production, a scaffold should promote myogenesis and possess good cytocompatibility. It is reported that the fibroblast-like morphology of myoblasts changes substantially to cluster when growing in 3D culture, which represents the early stages of in vitro myogenesis [33,35]. Likewise, myoblast cells seeded on DMS formed clusters that were characterized by histochemical staining with trichrome stain as trichrome stain imparts red color to the muscle cell cytoplasm [50]. Red-stained clusters of the myoblast cells growing on the scaffold demonstrate the myogenic supporting capacity of the DMS. In addition, gene expression of *MHC* in the myoblast cells in monolayer and clusters grown on DMS confirms that the scaffold supports the differentiation and early myogenesis of the myoblast cells.

The application of decellularized tissue scaffold is still in its infancy; however, it is being explored for various tissue-engineering applications. So far, various decellularized plant tissue scaffolds have been used for cultured meat production. The current decellularized plant scaffolds only provide a three-dimensional scaffold for cell growth, but their biological activity is poor [41]. Biodegradation of plant scaffolds is a serious concern; Lee and colleagues observed that plant-derived scaffolding implants remained intact after 8 weeks because animals lack appropriate enzymes to digest plant cellulose [51]. In addition, the existing decellularization protocols damage the structure of plants, including increasing pores and decreasing mechanical properties [41]. Therefore, the decellularization methods suitable for plants, especially for some fragile tissues, require refinement, and their applicability to tissue engineering needs to be further improved. To overcome the disadvantages associated with decellularized plant tissue scaffolds, decellularized mushroom scaffolds, which exhibit good scaffold properties and cytocompatibility, could be utilized for alternative animal protein production in the form of cultured meat.

The results clearly indicate that DMS displays good cytocompatibility and favors the myogenic differentiation of the myoblast cells and, thus, could be applied as an efficient source of alternative animal protein production. However, DMS has not been used with livestock satellite cells or muscle cells, which is the limitation of the study. Thus, further research on the assessment of cytocompatibility and differentiation of livestock satellite cells or myoblast with DMS is required.

## 5. Conclusions

Cultured meat, a prominent source of alternative animal protein, could help to remove the increasing prevalence of human diseases associated with conventional meat production and consumption. A decellularized tissue scaffold has been suggested as a potential scaffold for growing muscle tissue and up-scaling cultured meat production. Results display the good cytocompatibility of the DMS, which supports the growth of the viable myoblast cells and promotes the myogenesis of the differentiated myoblast cells. Moreover, DMS could be explored for alternative animal protein production as it not only shows good cytocompatibility and myogenesis induction properties but also possesses high nutritional value and degradability. DMS could further be used to assess the compatibility with livestock myotubes so that its potential to be used as an edible scaffold for cultured meat production can be evaluated properly.

## Figures and Tables

**Table 1:** Primer sequence.

Sr.	Gene	Primer Sequence
1.	*MyoG* F *MyoG* R	5′GCTCAAGAAAGTGAATGAGGC 3′ 5′ CTGGTAGACTCCTTCCTGCAG 3′
2.	*MHC* F *MHC* R	5′ GGCCAAATCAAAGAGGTGA 3′ 5′ CGTGCTTCTCCTTCTCAACC 3′
3.	*GAPDH* F *GAPDH* R	5′ GAAGGTCGGTGTGAACGGAT 3′ 5′ ATGAAGGGGTCGTTGATGGC 3′

## Data Availability

Data supporting the findings of this study are available from the corresponding author (VV) upon reasonable request.

## Supplementary Materials

The following supporting information can be downloaded at https://www.mdpi.com/article/10.3390/cells13010041/s1, Figure S1: Cytocompatibility of the DMS with goat dermal fibroblast cells; Figure S2: Cytocompatibility of the DMS with C2C12 myoblast cells at day 5; Figure S3: Viability assessment of the myoblast cells seeded on DMS. Calcein AM/PI staining of myoblast clusters on DMS at lower magnification.
