# Supplementary material for: Derivation and Characterization of Novel Cytocompatible Decellularized Tissue Scaffold for Myoblast Growth and Differentiation
Source: Cells. 2023 Dec 24;13(1):41. doi: 10.3390/cells13010041 (PMC10778107; doi:10.3390/cells13010041)
Supplement: Supplementary file 1 [file cells-13-00041-s001.zip › cells-2723339-supplementary.pdf]

Derivation and characterization of novel cytocompatible decellularized tissue scaffold for myoblast growth and differentiation

Anshuman Singh[1], Suraj Kumar Singh[1], Vinod Kumar[2], Manoj Kumar[3], Devojit Kumar Sarma[3], Samradhi Singh[3], Jalaj Gupta[1], Vinod Verma[1*]

[1]Stem Cell Research Centre, Department of Hematology, Sanjay Gandhi Post Graduate Institute of Medical Sciences, Lucknow, India

[2]National Institute of Animal Biotechnology (NIAB), Hyderabad, Telangana, India

[3]ICMR- National Institute for Research in Environmental Health, Bhopal, India

Supplementary data

The cytocompatibility of DMS with Goat dermal fibroblast cells

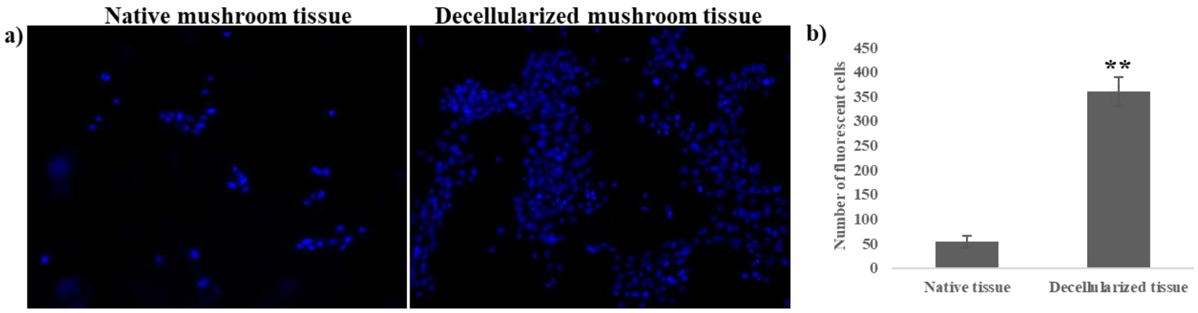

Supplementary Figure S1: Cytocompatibility of the DMS with goat dermal fibroblast cells. a) DAPI staining of the fibroblast cells seeded on native and decellularized mushroom tissue scaffold. b) Quantification showed a significantly higher number of cells attached to DMS in comparison to native tissue (p-value 0.005). Data are reported as mean ± SD. **P＜0.01.

Cytocompatibility of DMS with C2C12 myoblast cells at Day 5 (120hrs)

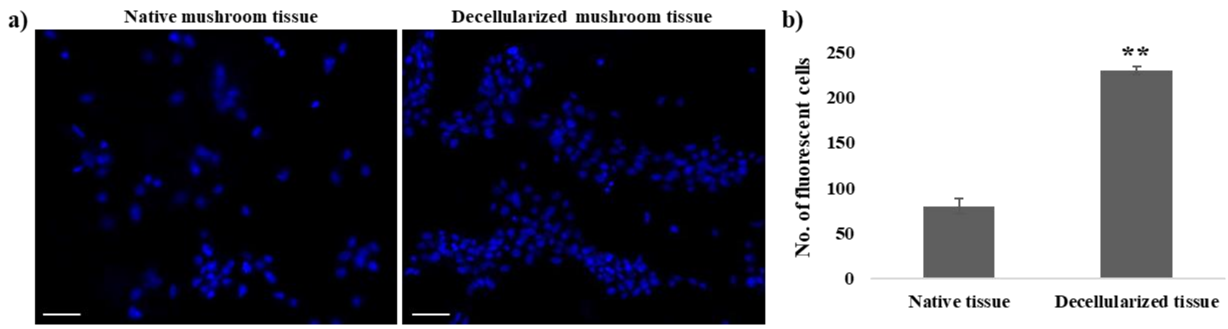

Supplementary Figure S2: Cytocompatibility of the DMS with C2C12 myoblast cells at day 5. a) DAPI staining of the C2C12 cells seeded on native and decellularized mushroom tissue scaffold. b) Quantification showed a significantly higher number of cells attached to DMS in comparison to native tissue (p-value 0.002). Data are reported as mean ± SD. **P＜0.01.

Viability of C2C12 myoblast clusters on DMS (lower magnification)

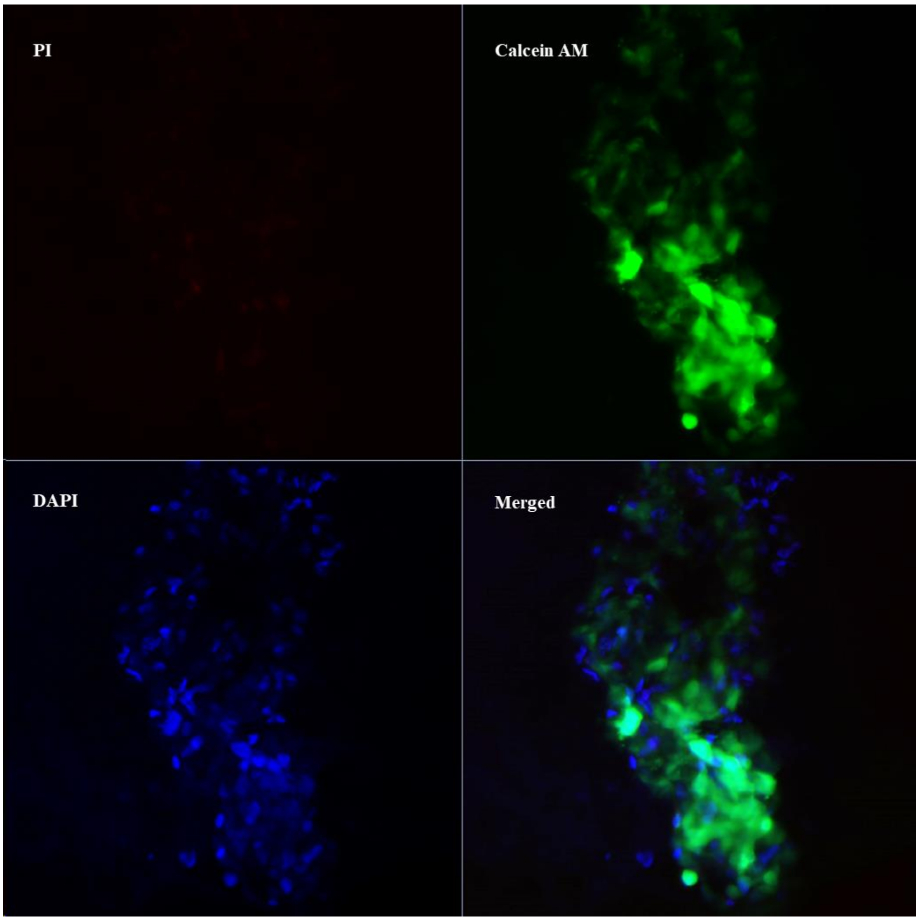

Supplementary Figure S3: Viability assessment of the myoblast cells seeded on DMS. Calcein AM/PI staining of myoblast clusters on DMS at lower magnification.
